# Acute and Subchronic Oral Toxicity Evaluation of Aqueous Root Extract of* Dicoma anomala* Sond. in Wistar Rats

**DOI:** 10.1155/2016/3509323

**Published:** 2016-04-13

**Authors:** Fatai Oladunni Balogun, Anofi Omotayo Tom Ashafa

**Affiliations:** Phytomedicine and Phytopharmacology Research Group, Department of Plant Sciences, University of the Free State, Qwaqwa Campus, Private Bag X13, Phuthaditjhaba, Free State 9866, South Africa

## Abstract

The present study evaluated the safety of aqueous root extract of* Dicoma anomala* (AQRED) through acute and subchronic toxicity studies. Single oral dose of AQRED at the concentration of 0, 5, 300, and 2000 mg/kg as well as 125, 250, and 500 mg/kg/day was administered to rats for 14-day acute and 90-day subchronic oral toxicity studies. The results revealed no mortalities or observed clinical signs of toxicity in all the rats during both investigation periods. In subchronic toxicity testing, administration of AQRED also did not cause any changes in body weight as well as food and water consumption patterns. The haematological parameters and blood chemistry revealed no significant difference (*p* > 0.05) between the treatment and the control except in platelet count, alkaline phosphatase, and sodium levels where there was a significant increase (*p* < 0.05), although there was also a significant reduction (*p* < 0.05) in alanine transaminase, aspartate transaminase, and creatinine when compared to control. However, these changes were not reflecting the results from histology. Conclusively, the obtained results suggested that the LD_50_ of AQRED is in excess of 2000 mg/kg and its oral administration for 90 days revealed that it is unlikely to be toxic, hence, safe.

## 1. Introduction

*Dicoma anomala* belongs to the family of Asteraceae and is locally called Hloenya (South Sotho) or fever or stomach bush (Afr.). This herbaceous plant which grows in grasslands or stony places has erected stems, thinly covered with hairs, and originates from a woody rootstock. The leaves are simple, alternate, stalkless, linear, or narrowly lanceolate. The flower heads are terminal, solitary or in small pairs, narrow, with sharply pointed bracts and slender, with white, mauve, purple, or pink tubular florets [1].* D. anomala* is widely distributed in sub-Saharan Africa including most provinces such as Limpopo, North West, Gauteng, Mpumalanga, Free State, Northern Cape, and KwaZulu-Natal within South Africa [1]. The plant is traditionally used for the treatment of cold and coughs, fever, ulcers, and dermatosis mostly among the Basotho tribe of eastern Free State. A number of documented pharmacological potentials such as antiplasmodial, antibacterial, anthelmintic, antiviral, and anti-inflammatory have been reported [2]. However, despite the various pharmacological efficacies in relation to numerous health-related benefit of the plant, it is noteworthy not to rule out issues of safety. Moreover, based on documented reports, continuous intake of high dose of medicinal plants and/or associated chemical compounds could alter hepatic and thyroid function adversely [3–6] and to date the potential toxicities of* Dicoma anomala* have not gotten enough attentions when taken in increased concentration despite the recently reported antioxidant activity of this plant by our research group. In this study, we evaluated the toxicity of the aqueous root extract with the dietary administration to male and female Wistar rats for acute 14 days and subchronic 90 days as parts of a safety assessment according to the internationally acceptable guidelines. We also subjected the extract to gas chromatography/mass spectrophotometric (GC/MS) analysis for possible identification of the phytoconstituents present in the plant.

## 2. Materials and Methods

### 2.1. Plant Collection and Extraction

Fresh root stocks of* D. anomala* were procured in April 2014 from Setsing market, Phuthaditjhaba, Qwaqwa, Free State province, South Africa. The sample was confirmed by Dr. AOT Ashafa of Plant Sciences Department, University of the Free State, Qwaqwa Campus, Free State, and a voucher specimen with (BalMed/01/2015/QHB) was thereafter prepared and deposited at the Department of Plant Sciences herbarium. A total of 5.2 kg of the root sample was cut into smaller pieces, oven-dried (40°C) for 96 hr, and pulverized using a Waring Commercial blender (USA) to yield 3.063 kg fine powder. Approximately, 600 g of the powdered root material was extracted with 5 L of distilled water on a platform shaker for 5 days. The resulting mixture was centrifuged at 1285 g for 5 min using a BHG Roto-Uni II centrifuge and afterwards filtered using Whatman number 1 filter paper. The filtrates obtained were pooled together and concentrated to dryness on a water bath (Memmert W600, Schwabach, Germany) at 40°C. The extraction yielded 137.27 g of brown gum (22.88% w/w of dry plant material) crude extract. The crude extract was reconstituted in water to afford various concentrations used in this study.

### 2.2. Experimental Animals

A total of one hundred (both sexes) 5-week-old Wistar rats were purchased from animal facility of University of the Free State, Bloemfontein, and were used after a week of acclimatization. Individual body weights were recorded and detailed physical examinations were performed twice during the acclimation period to ensure the use of healthy animals. Animals were housed in clean metabolic cages placed in a ventilated house and allowed free access to commercial laboratory feed (Epol rat cubes, South Africa) and tap water* ad libitum*. Animals were subjected to housing condition of the controlled system of the light-dark cycle (12-12 h), ventilation (air-exchange rate of 18 times per hour), temperature (23 ± 2°C), and relative humidity (55 ± 5%) during the study. The cages and the chip bedding were exchanged twice weekly and feed intake and water consumption, on a daily basis (by measuring the left-over feed/water and subtracting it from the initial weight or volume) and body weight measurement was taken on a weekly basis. Ethical approval for use of animals was obtained from Interfaculty Ethics Committee of the University of the Free State (NR/02/13) prior to the commencement of the study and the study was performed in accordance with the stipulation for the care and use of laboratory animals, prepared by National Institute of Health, USA (Guide for the Care and Use of Laboratory Animals, 1996).

### 2.3. Acute Oral Toxicity Study of Aqueous Root Extract of* D. anomala* (AQRED)

The single dose acute oral toxicity study was evaluated following the recommendations by The Organisation for Economic Co-operation and Development (OECD, 2001) [7]. Animals (female rats) were randomly divided into four groups of 5 animals. After overnight fasting (i.e., withdrawal of feeds only and not water for about 12–15 h), they were administered a single oral dose of 0, 5, 300, and 2000 mg/kg body weight AQRED by oral gavage. Distilled water was administered to the control group. Feeding was commenced 4 h after the administration and all animals were observed for clinical signs including mortality and moribundity immediately at 1, 2, 4, 8, and 12 h and then twice daily until day 14. Body weights, food consumption, and water intake were measured on days 0, 1, 5, 10, and 14. On the 14th day, all animals were sacrificed and all organs and tissues were observed macroscopically. The organs were fixed in 10% neutral buffered formalin and observed for histopathological examination.

### 2.4. Subchronic 90-Day Oral Toxicity Study of AQRED

#### 2.4.1. Experimental Design

The subchronic 90-day oral toxicity study was evaluated following the recommendations by OECD (1998) [8]. Four groups of ten (10) male and female Wistar rats received doses of 0, 125, 250, and 500 mg/kg body weight (BW) of AQRED at daily gavage of 1 mL/100 g (BW) for 90 consecutive days. Observations were made twice daily for mortality and changes in general appearance or behaviour. The body weights were recorded every week and the individual dose administered to the animals was adjusted weekly for the body weight to maintain the target dose level for all rats. Additionally, detailed clinical examination and measurement of food and water consumption were made daily.

#### 2.4.2. Blood Preparation and Organ Isolation

The animals were anaesthetized with halothane at the end of the experimental period (i.e., day 90 of treatment) and blood was collected by clearing the neck region of fur to locate the jugular vein. An aliquot (2 mL) of the blood was collected into ethylene diamine tetra-acetic acid (EDTA) bottle and was used for the analysis of haematological parameters, while another 5 mL of the blood collected in nonheparinized bottle was centrifuged at 1285 g for 10 minutes; the resulting serum was aspirated and used for other serum bioassays. The animals were quickly dissected and the liver, kidney, heart, lungs, spleen, stomach, testes, ovary, brain, and pancreas were excised, freed of fat, blotted with clean tissue paper, and weighed for evaluation of organ-to-body weight ratios. Defined samples of the liver, brain, pancreas, kidney, and testes were placed in 10% neutral buffered formalin for histopathological examination. All organs were visually inspected and weighed directly after dissection to reduce mechanical damage.

#### 2.4.3. Hematology and Blood Chemistry

One blood sample (approximately 20 *μ*L) was treated with EDTA-2K for white blood cells (WBC), red blood cells (RBC), haemoglobin (Hb), platelets (PLT), percent of lymphocytes (LY), percent of monocytes (MO), haematocrit (HCT), mean corpuscular haemoglobin (MCH), mean corpuscular haemoglobin concentration (MCHC), mean corpuscular volume (MCV), mean platelet volume (MPV), platelet haematocrit (PCT), platelet distribution width (PDW), and red blood cell distribution width (RDW) using a hematology analyzer MEK-6318K (Nihon Kohden Co., Ltd.). Serum from blood samples collected in separator tubes were measured using a BS-200 automatic biochemistry analyzer (Mindray Co., Ltd.) including aspartate aminotransferase (AST), alanine aminotransferase (ALT), urea, nitrogen, creatinine (Cr), total cholesterol (TC), triglyceride (TG), total protein (TP), albumin (Alb), and glucose (Glu). Total calcium (TCa) was measured using the 7020 automatic biochemistry analyzer (Hitachi Co., Ltd.) as well as sodium (Na), potassium (K), and chloride (Cl).

#### 2.4.4. Histopathological Studies

Pieces of vital organs from each group were fixed immediately in 10% neutral formalin for a period of at least 24 h, dehydrated in graded (50–100%) alcohol, cleaned with xylene, embedded in paraffin, cut into 4-5 *μ*m thick sections, and stained with hematoxylin-eosin. The microscopic features of the organs of male and female rats were compared with that of the control group.

#### 2.4.5. Statistical Analysis

Statistical analysis was performed using GraphPad Prism 5 statistical package (GraphPad Software, San Diego, CA, USA). Data were expressed as means of ten replicates ± standard error of mean (SEM) for all assays and were subjected to one-way analysis of variance (ANOVA) followed by Dunnett's multiple comparison test. Statistical significance was considered at *p* < 0.05.

### 2.5. Gas Chromatography/Mass Spectrophotometric Analysis

The GC/MS analysis of the extract was performed on a QP-2010 system (Shimadzu, Japan) via electron ionization (EI) at 70 eV. GC separation was achieved on a capillary column (0.25 mm i.d × 30 m, film 0.5 mm) of HP-5MS (Hewlett Packard, USA). The injection of the extract (1 mL) was done using the splitless injection method. The initial column temperature was 50°C, then went up to 250°C at a rate of 10°C/min, and finally held at 250°C for 5 min. The injection port was set at 120°C. Helium gas was used as the carrier at a flow rate of 0.6 mL/min [9]. The mass spectra of the constituents were compared with known spectra stored in Wiley library.

## 3. Results

### 3.1. Single Dose Subacute Oral Toxicity Study of AQRED in Wistar Rats

No deaths occurred during the 14-day study. There were no significant differences in body weights and food consumption of rats between the AQRED administrated group and the controls (data not shown). A short time of sluggish movement appeared in some AQRED treated rats immediately after oral gavage at 2000 mg/kg body weight concentration in the first hour, but the rats continued to live afterwards. Aside from this observation, there were no abnormal findings of other clinical signs and autopsy performed in all the experimental and control animals. It was based on these findings that the LD_50_ value of AQRED was assumed to be greater than 2000 mg/kg since no mortality or signs of toxicity were observed in all experimental animals.

### 3.2. Subchronic 90-Day Oral Toxicity Study of AQRED in Wistar Rats

#### 3.2.1. Clinical Signs and Mortality

There was no mortality attributed to any effect of AQRED during the 90-day administration. Similarly, there was no treatment-related change at the autopsy in the AQRED groups or control group.

#### 3.2.2. Feed and Water Intake

**Figure 1:**
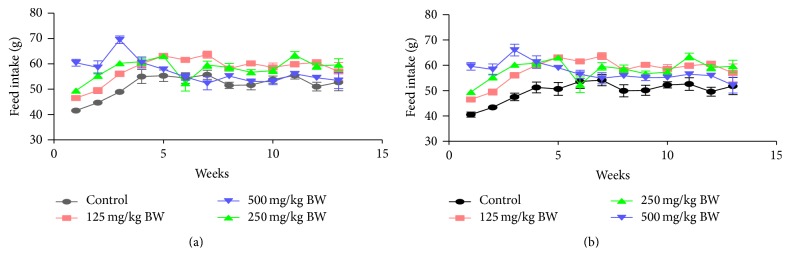
Effect of aqueous root extract of* Dicoma anomala* on food consumption for male and female Wistar rats during a 90-day subchronic toxicity study. Values were calculated and data expressed as mean ± (standard error of mean) SEM [BW: body weight].

The effect of aqueous root extract of* Dicoma anomala* on feed intake is shown in Figures 1(a) and 1(b). From the results, it was observed that, during the 90-day study, there were no significant differences (*p* > 0.05) in mean food consumption among all the experimental animals. There seems to be an increase in feed intake as the experimental days increased.

**Figure 2:**
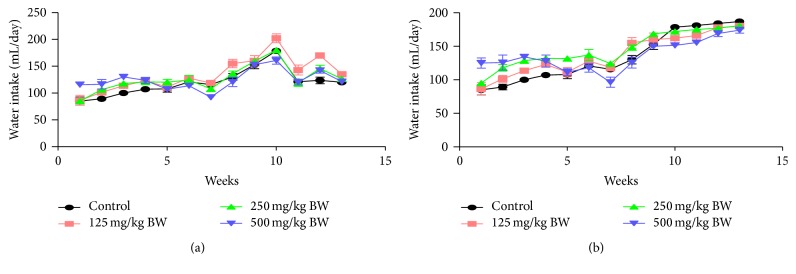
Effect of aqueous root extract of* Dicoma anomala* on water intake of male and female Wistar rats during a 90-day subchronic toxicity study. Values were calculated and data expressed as mean ± SEM.

The result of the effect of aqueous extract of* Dicoma anomala* on water intake (Figures 2(a) and 2(b)) revealed no significant difference (*p* > 0.05) in water intake during the 13-week study for both sexes of the animals.

#### 3.2.3. Organ Weights

The results of relative organ weight of the animals are shown in Tables 1 and 2. It was observed that there was no statistical difference (*p* > 0.05) in all the excised organs as compared with the control group.

#### 3.2.4. Body Weights

**Figure 3:**
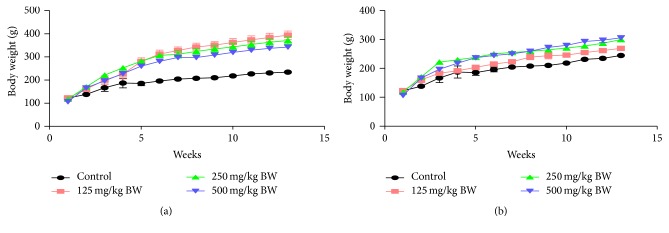
Effects of oral administration of aqueous root extracts of* Dicoma anomala* on mean body weights of male and female Wistar rats. Body weight was calculated and data expressed as mean ± SEM.

The mean body weights of the animals at all concentrations were compared to the values of control (Figures 3(a) and 3(b)). The rats in all AQRED groups had statistically (*p* < 0.05) higher mean body weights (394.20 g, 371.10 g, and 342.60 g in 125, 250, and 500 mg/kg/day group, resp.) of males as compared to the control group (234.50 g). Moreover, there was a significant difference (*p* < 0.05) in body weight of female rats at all the three concentrations 125, 250, and 500 mg/kg body weight (269 g, 299 g, and 306 g, resp.) compared to control (244 g).

#### 3.2.5. Haematology and Blood Chemistry

The results from haematological parameters only revealed statistically significant decrease (*p* < 0.05) in platelet count compared with control groups for both sexes, while there was no significant difference in other parameters compared to the control groups (Tables 3 and 4). Moreover, the result obtained from the blood chemistry showed a dose-dependent decrease (*p* < 0.05) in creatinine level and alanine transaminase activity, while it recorded a dose-dependent increase (*p* < 0.05) in sodium level. Serum activity of alkaline phosphatase also showed a statistical increase (*p* < 0.05) in the entire dose regimen, while the reverse is the case for aspartate transaminase when compared with control groups. Beyond all the reported results, there was no treatment-related or statistically significant (*p* < 0.05) effects observed in all the other tested blood chemistry parameters (Tables 5 and 6).

#### 3.2.6. Histopathological Examination

The result from histological analysis revealed no macroscopic observations considered to be treatment-related. No gross deformities could be considered to be related to the test article for any of the euthanized animals necropsied at the expiration of the 90-day experimental study. Histopathological observation including presence of hyperplasia and lymph nodes was only noticeable in one of the sections of the stomach for the 125 mg/kg body weight male rats as well as presence of renal calculi in the collecting tubes of the kidney for untreated control animals; as such, these changes could not be treatment-related.

### 3.3. GC/MS Analysis

The GC/MS chromatogram of* Dicoma anomala* extract revealed the presence of rich amounts of eighty phytochemical compounds. The major identifiable constituents are 5-(Hydroxymethyl)-2-furancarboxyaldehyde, 2-(1-methyl-2,5-dioxoimidazolidin-4-yl) acetic acid, n-tridecane, 9-octadecenoic acid, hexadecanoic acid, phosphine, 1,6-anhydro-*β*-D-glucopyranoside, 3-(Bromophenyl) triphenyl phosphonium bromide, Guanosine, and Xanthosine. The compound name, molecular formula, and peak area (%) of the majority of the constituents are presented in Table 7.

## 4. Discussion

The recognition of herbal treatment or phytomedicine as the most common form of alternative medicine has been around since time immemorial [26]. This is because a larger percentage of the world's population (about 80% according to World Health Organization's estimation) depends on these plant-based remedies as a viable option to diseased conditions most especially in developing and/or developed countries where conventional or modern drugs are majorly used [27]. Similarly, it is worth mentioning that the popularity, as well as the usage of these traditional medicines, has continued to increase all over the world [28]. Despite this popularity and wide usage, the safety of these herbal therapies has, in recent times, raised a lot of questions as a result of revelations due to illnesses and fatalities [29, 30] such as hepatotoxicity [31] and nephrotoxicity [32, 33] and only a few of them have been evaluated through various phases of clinical trials [34].

Toxicity studies encompass acute, subacute, chronic, or subchronic toxicity. In the present study, acute and subchronic toxicities studies were evaluated on* Dicoma anomala* aqueous root extract that paved the way for the meaningful interpretation of its toxic effect. The single oral dose administration of AQRED to Wistar albino rats at the highest concentration of 2000 mg/kg body weight did not cause any mortality and revealed no clinical signs, like changes in fur and skin, eyes, mucus membrane, respiratory rate, circulatory signs, autonomic effects, and central nervous system. In general, exposure of the animals to a single oral dose of AQRED did not produce any mortality or any treatment-related effect [35]; as such, the lethal dose (LD_50_) was assumed to be greater than 2000 mg/kg suggesting that the extract could be generally regarded as nontoxic. This report corroborates the work of M. L. Clarke and E. G. C. Clarke [36] who maintained that any drug with oral LD_50_ greater than 1000 mg/kg BW could be regarded as safe and of low toxicity.

The administration of AQRED for 90 days revealed no clinical signs of toxicity or mortality in both sexes of the animals used in this study. There was no significant reduction in feed and water intake of the treated rats in either sex through the 90-day study; this is a pointer to the fact that the diet and water were well accepted by the rats, suggesting that the extracts did not in any way alter the metabolism of carbohydrate, protein, and fats in the rats. It may also signify the fact that the nutritional status (weight gain and appetite stability) which was expected to be seen in prolonged-fed animals was not adversely affected by the extracts. This corroborates the traditional usage of the plant by the oral route.

The body weight changes may reflect the general health status of animals [37]. However, the body weight gain witnessed in all the animals treated with AQRED suggests that the extracts did not interfere with normal body metabolism of the animals as the increment in food and water intake is synonymous to an increase in body weight. The essence of weighing organs in studies relating to toxicity provides facts on their sensitivity to toxicity, physiologic perturbations, induction of enzymes, and acute organ damage [38]. An insignificant difference in the weight of the excised vital organs compared to that of the control obtained for this study was an indication that AQRED on prolonged use or intake might not have an effect on normal growth; and since there was no reduction in body and/or relative organ weight in all the tested doses of the treated rats, it is assumed that the extract is not toxic to the excised and evaluated organs.

The assessment of blood hematology and clinical biochemistry provides an insight to possible damage brought about by the extract in the hepatic and renal functions. In toxicity studies, assessment of liver and kidney functions is germane because both organs are essential for the survival of an organism [39]. Alanine aminotransferase (ALT), aspartate transaminase (AST), and alkaline phosphatase (ALP) are sensitive enzymes used in assessing the severity of liver damage [40]. Elevated activities of these enzymes are associated with liver or heart damage [41–43]. The significant reduction in ALT and AST of the treated animals in both sexes compared with normal control could suggest that AQRED may not have hepatotoxic effect [44] and, equally, might not have deleterious effect on the heart [45], while the increase in ALP might suggest obstruction of the biliary tract which may be present in the liver. Evaluation of total protein gives an estimation of the nutritional status and diagnostic measurement of liver and kidney diseases [46]. A reduction in total protein, albumin, and globulin is an indication of impaired hepatocellular function [47]; however, an insignificant difference in serum level of these parameters upon prolonged administration of* D. anomala* when compared with the control further corroborates the fact that the extracts do not destroy the secretory functions of the liver. Measurement of serum urea, creatinine, and uric acid concentration reflects the likelihood of renal problems or dysfunction [48]. The insignificant changes in urea and the significant reduction in creatinine levels obtained in this study as compared to the normal control could suggest an indication of the potentials of AQRED in maintaining a normal renal function [45]. The result was in consonance with the report of Patrick-Iwuanyanwu et al. [49] for Baker cleansers bitters.

Abnormalities in the concentration of major lipids like cholesterol (TC), high-density lipoprotein cholesterol (HDL-c), and triglycerides (TG) can give useful information on the lipid metabolism as well as the predisposition of the animals to atherosclerosis and its complications. The insignificant changes in the value of TC, LDL-c, HDL-c, and triglycerides recorded in this study might signify the intact position of the myocardial membrane [50] and could also suggest the hypolipidemic potentials of the plant [51].

A prominent role in the exchange of gas and the intercompartmental water balance is played by electrolytes. Increase or decrease in the levels of these electrolytes within the serum may be a consequence of the hypo- or hyperfunctioning of the concerned organ or tissue. Sodium, potassium, and chlorides are examples of these clinical electrolytes used in assessing the functioning of the kidney. In the present study, there was no significant change in the treated rats in these parameters when compared with the control; this could be attributed to a normal functioning status of the kidney.

Haematological evaluation is used to determine the extent of the deleterious effect on the blood. It can also explain the blood relating functions of a plant extract or its products [52]. The result from the present study revealed no significant effects on red blood cells (RBC), MCV, and haemoglobin values of the treated rats when compared to the control, suggesting that the erythropoiesis, morphology, or osmotic fragility of RBC are not affected [53]. Similarly, the insignificant changes in neutrophils, lymphocytes, and monocytes witnessed in all the tested concentrations similarly suggested the intact state of the immune system and lack of injury to the tissues, while the significant increase in platelets concentration for all the treated groups compared to control may be attributed to the elevated secretion and production of thrombopoietin, the primary regulator of platelet production [54], by AQRED suggesting its homeostatic property [55]. This result was in line with the report of Patrick-Iwuanyanwu et al. [49]. Generally, the result obtained from the hematology examinations further justified the safety potential of AQRED.

The histological examination of all the analysed (vital) organs revealed that none of the organs from the extract treated rats revealed alteration and there were no abnormalities in the cell structure of these vital organs.

Besides being antioxidative [15, 20, 21], most of the identified compounds from the GC/MS chromatogram of the plant have been implicated in other biological and pharmacological activities. 5-(Hydroxymethyl)-2-furancarboxyaldehyde, 2,3-dihydro-3,5-dihydroxyl-6-methyl-4H-pyran-4-one, n-tridecane, and globulol are reported to possess antimicrobial activity [10, 15, 16, 22, 23]. The presence of 5-(Hydroxymethyl)-2-furancarboxyaldehyde and 2,3-dihydro-3,5-dihydroxyl-6-methyl-4H-pyran-4-one in the phytonutrients is attributed to their anti-inflammatory potentials [11–13, 15]. 2-(1-methyl-2,5-dioxoimidazolidin-4-yl) acetic acid and 5-(Hydroxymethyl)-2-furancarboxyaldehyde have been reported to have antihepatotoxic effect [14, 18]. Some of the other pharmacological activities such as anticancer [20, 21] and antihypertensive [25] exhibited by the phytonutrients are shown in Table 7.

## 5. Conclusions

In conclusion, the administration of aqueous root extract of* Dicoma anomala* to Wistar rats was not toxic in all the tested doses and did not reveal any toxic signs in the two studied toxicities. The extract did not have a direct impact on the liver and kidney functions as evidenced from the haematological and blood chemistry results and did not cause any change in food intake, water consumption, and body weight and produces no evident histopathological damage in the rats. The presented results might give an insight to exploring the pharmaceutical and therapeutic benefit of AQRED as a viable candidate to conventional drugs with lots of side effects. Moreover, the presence of various bioactive compounds as revealed by the GC/MS result and the confirmation of some of the therapeutic properties justify the usage of the plants for various ailments by traditional healers. Further studies to ascertain the effects of the herb on pregnant animals, animal foetus, and their reproductive capability are however encouraged to fully explore the safety profile of the plant.

## Figures and Tables

**Table 1:** Effect of oral administration of aqueous extract of *Dicoma anomala* for 90 days on relative organ-to-body weight ratio of male Wistar rats.

Organ	Dose (mg/kg/day)			
	Control	125	250	500
Initial weight^a^	122.60 ± 2.55	123.00 ± 2.94	117.60 ± 4.09	106.60 ± 5.17
Body weight^a^	234.50 ± 6.92	394.20 ± 17.77^*∗*^	371.10 ± 15.08^*∗*^	342.60 ± 4.70^*∗*^
% weight change	91.27	220.48	215.56	221.39
Liver^a^	5.27 ± 0.19	9.63 ± 0.95	10.22 ± 0.23	8.22 ± 0.39
Brain^a^	1.23 ± 0.05	1.35 ± 0.05	1.28 ± 0.22	3.17 ± 0.16
Heart^a^	0.82 ± 0.03	1.42 ± 0.03	1.42 ± 0.06	1.22 ± 0.12
Lungs^a^	2.02 ± 0.36	3.26 ± 0.62	3.39 ± 0.32	1.95 ± 0.20
Kidney^a^	1.69 ± 0.18	2.45 ± 0.28	2.45 ± 0.10	2.22 ± 0.16
Spleen^a^	0.46 ± 0.03	0.62 ± 0.08	0.69 ± 0.02	0.60 ± 0.07
Stomach^a^	2.65 ± 0.12	2.94 ± 0.44	3.02 ± 0.52	3.17 ± 0.05
Testes^a^	3.16 ± 0.12	4.58 ± 1.16	3.41 ± 0.21	3.33 ± 0.25
Pancreas^a^	0.96 ± 0.03	1.52 ± 0.01	1.64 ± 0.26	1.36 ± 0.18
Liver^b^	2.25	2.44	2.75	2.4
Brain^b^	0.52	0.34	0.34	0.93
Heart^b^	0.35	0.36	0.38	0.36
Lungs^b^	0.86	0.83	0.91	0.57
Kidney^b^	0.72	0.62	0.66	0.65
Spleen^b^	0.20	0.16	0.19	0.18
Stomach^b^	1.13	0.75	0.81	0.93
Testes^b^	1.35	1.16	0.92	0.97
Pancreas^b^	0.41	0.39	0.44	0.40

Mean organ-to-body weight ratio was calculated and expressed as mean ± SEM (*n* = 10).

^a^Unit: g.

^b^Unit: % body weight.

^*∗*^Statistically significant compared to control (*p* < 0.05).

**Table 2:** Effect of oral administration of aqueous extract of *Dicoma anomala* for 90 days on relative organ-to-body weight ratio of female Wistar rats.

Organ	Dose (mg/kg/day)			
	Control	125	250	500
Initial weight^a^	120.60 ± 2.55	121.00 ± 2.94	119.60 ± 4.09	116.60 ± 5.17
Body weight^a^	244.50 ± 6.94	269.20 ± 0.76	299.40 ± 3.22	306.00 ± 4.91^*∗*^
% weight change	102.74	122.48	150.33	162.44
Liver^a^	6.87 ± 0.19	5.99 ± 0.95	6.29 ± 0.23	6.22 ± 0.32
Brain^a^	1.01 ± 0.05	1.15 ± 0.05	1.23 ± 0.21	1.17 ± 0.16
Heart^a^	0.52 ± 0.03	0.62 ± 0.03	0.58 ± 0.06	0.60 ± 0.12
Lungs^a^	1.52 ± 0.36	1.36 ± 0.62	1.39 ± 0.12	1.45 ± 0.20
Kidney^a^	1.39 ± 0.18	1.40 ± 0.28	1.45 ± 0.10	1.35 ± 0.06
Spleen^a^	0.32 ± 0.03	0.40 ± 0.08	0.39 ± 0.02	0.41 ± 0.07
Stomach^a^	2.05 ± 0.12	2.44 ± 0.44	2.12 ± 0.52	2.17 ± 0.05
Ovary^a^	3.16 ± 0.12	4.58 ± 1.16	3.41 ± 0.21	3.33 ± 0.25
Pancreas^a^	0.76 ± 0.03	0.78 ± 0.01	1.64 ± 0.26	1.36 ± 0.18
Liver^b^	2.81	2.23	2.10	2.03
Brain^b^	0.41	0.43	0.41	0.38
Heart^b^	0.21	0.23	0.19	0.20
Lungs^b^	0.62	0.51	0.46	0.47
Kidney^b^	0.57	0.52	0.48	0.44
Spleen^b^	0.13	0.15	0.13	0.13
Stomach^b^	1.84	0.91	0.71	0.71
Ovary^b^	1.29	1.70	0.14	1.09
Pancreas^b^	0.31	0.29	0.55	0.44

Mean organ-to-body weight ratio was calculated and expressed as mean ± SEM (*n* = 10).

^a^Unit: g.

^b^Unit: % body weight.

^*∗*^Statistically significant compared to control (*p* < 0.05).

**Table 3:** Haematological data for male rats given aqueous root extracts of *Dicoma anomala *for 90 days.

Parameters	Unit	Dose (mg/kg/day)			
		Control	125	250	500
RBC	millions/*µ*L	8.42 ± 0.10	9.44 ± 0.01	9.3 ± 0.05	9.18 ± 0.02
Haemoglobin	g/dL	16.4 ± 0.01	17.6 ± 0.02	16.9 ± 0.01	16.9 ± 0.01
Haematocrit	L/L	0.55 ± 0.05	0.61 ± 0.01	0.58 ± 0.02	0.59 ± 0.00
MCV	fL	65.00 ± 0.00	64.00 ± 0.00	62.00 ±0.00	64.00 ± 0.00
MCH	pg	19.00 ± 0.01	19.00 ± 0.01	18.00 ± 0.01	19.00 ± 0.01
MCHC	g/dL	30.00 ± 0.10	29.00 ± 0.10	29.00 ± 0.10	29.00 ± 0.10
RDW	fL	12.60 ± 0.01	13.40 ± 0.10	14.00 ± 0.00	14.10 ± 0.2
WBC	thous/*µ*L	5.80 ± 0.02	6.50 ± 0.01	6.40 ± 0.02	6.90 ± 0.02
Neutrophils	×10^9^/L	0.22 ± 0.00	0.46 ± 0.01	0.51 ± 0.02	0.62 ± 0.01
Lymphocytes	%	4.58 ± 0.10	4.45 ± 0.20	4.37 ± 0.20	4.89 ± 0.10
Monocytes	%	0.92 ± 0.00	1.43 ± 0.01	1.38 ± 0.01	1.28 ± 0.02
Eosinophils	×10^9^/L	0.07 ± 0.00	0.16 ± 0.02	0.09 ± 0.00	0.08 ± 0.00
Basophils	×10^9^/L	0.01 ± 0.00	0.02 ± 0.00	0.03 ± 0.01	0.02 ± 0.01
Platelet	thous/L	979.00 ± 0.50	803.00 ± 0.03^*∗*^	701.00 ± 0.10^*∗*^	754.00 ± 0.49^*∗*^

Values are presented as mean ± SEM (*n* = 10).

^*∗*^Significantly different compared to control at *p* < 0.05.

**Table 4:** Haematological data for female rats given aqueous root extracts of *Dicoma anomala *for 90 days.

Parameters	Unit	Dose (mg/kg/day)			
		Control	125	250	500
RBC	millions/*µ*L	7.45 ± 0.10	7.44 ± 0.01	7.53 ± 0.05	7.88 ± 0.02
Haemoglobin	g/dL	14.41 ± 0.01	13.96 ± 0.02	15.09 ± 0.01	15.19 ± 0.01
Haematocrit	L/L	0.50 ± 0.05	0.49 ± 0.01	0.56 ± 0.02	0.59 ± 0.00
MCV	fL	61.00 ± 0.00	60.00 ± 0.00	62.00 ±0.00	63.00 ± 0.00
MCH	pg	16.00 ± 0.01	17.00 ± 0.01	17.00 ± 0.01	18.00 ± 0.01
MCHC	g/dL	27.00 ± 0.10	27.00 ± 0.10	28.00 ± 0.10	28.00 ± 0.10
RDW	fL	10.60 ± 0.01	11.40 ± 0.10	11.09 ± 0.00	11.91 ± 0.2
WBC	thous/*µ*L	5.80 ± 0.02	6.30 ± 0.01	6.70 ± 0.02	6.91 ± 0.02
Neutrophils	×10^9^/L	0.12 ± 0.00	0.16 ± 0.01	0.15 ± 0.02	0.16 ± 0.01
Lymphocytes	%	4.08 ± 0.10	4.05 ± 0.20	4.07 ± 0.20	4.80 ± 0.10
Monocytes	%	0.73 ± 0.00	0.93 ± 0.01	1.08 ± 0.01	1.12 ± 0.02
Eosinophils	×10^9^/L	0.09 ± 0.00	0.12 ± 0.02	0.08 ± 0.00	0.08 ± 0.00
Basophils	×10^9^/L	0.01 ± 0.00	0.02 ± 0.00	0.03 ± 0.01	0.02 ± 0.01
Platelet	thous/L	1007.00 ± 0.50	893.00 ± 0.03^*∗*^	871.00 ± 0.10^*∗*^	944.00 ± 0.49^*∗*^

Values are presented as mean ± SEM (*n* = 10).

^*∗*^Significantly different compared to control at *p* < 0.05.

**Table 5:** Effect of aqueous root extracts of *Dicoma anomala* for 90 days on biochemical parameters of male Wistar rats.

Parameters	Unit	Dose (mg/kg/day)			
		Control	125	250	500
Total cholesterol	mmol/L	2.50 ± 0.23	2.10 ± 0.15	2.20 ± 0.13	2.00 ± 0.20
Triglycerides	mmol/L	0.65 ± 0.00	1.21 ± 0.11	0.87 ± 0.20	0.86 ± 0.01
HDL-c	mmol/L	1.60 ± 0.23	1.30 ± 0.17	1.40 ± 0.25	1.30 ± 0.19
LDL-c	mmol/L	0.90 ± 0.00	0.80 ± 0.11	0.80 ± 0.14	0.70 ± 0.50
ALT	U/L	51.00 ± 0.85	48.00 ± 0.55^*∗*^	47.00 ± 0.77^*∗*^	47.00 ± 0.74^*∗*^
AST	U/L	215.00 ± 0.85	188.00 ± 0.90^*∗*^	145.00 ± 0.87^*∗*^	192.00 ± 0.88^*∗*^
ALP	U/L	185.00 ± 0.87	200.00 ± 0.90^*∗*^	264.00 ± 0.47^*∗*^	220.00 ± 0.75^*∗*^
Glucose	mg/dL	4.23 ± 0.10	3.70 ± 0.11	4.27 ± 0.05	4.47 ± 0.23
Sodium	mmol/L	137.00 ± 0.67	141.00 ± 0.85^*∗*^	142.00 ± 0.44^*∗*^	142.00 ± 0.53^*∗*^
Potassium	mmol/L	5.40 ± 0.01	5.20 ± 0.01	4.80 ± 0.02	5.60 ± 0.00
Chloride	mmol/L	104.00 ± 0.11	103.00 ± 0.21	104.00 ± 0.12	104.00 ± 0.01
Calcium	mmol/L	2.38 ± 0.01	2.31 ± 0.10	2.32 ± 0.14	2.34 ± 0.20
Magnesium	mmol/L	0.99 ± 0.40	0.87 ± 0.02	0.95 ± 0.14	0.88 ± 0.01
Urea	mmol/L	8.10 ± 0.01	7.50 ± 0.00	7.90 ± 0.12	7.50 ± 0.24
Creatinine	*µ*mol/L	44.00 ± 0.23	41.00 ± 0.21^*∗*^	40.00 ± 0.17^*∗*^	35.00 ± 0.20^*∗*^
Albumin	g/L	34.00 ± 0.15	32.00 ± 0.20	31.00 ± 0.10^*∗*^	32.00 ± 0.05
Total protein	g/L	65.00 ± 0.3	65.00 ± 0.25	63.00 ± 0.33	64.00 ± 2.0
Total bilirubin	*µ*mol/L	3.00 ± 0.11	3.00 ± 0.12	2.00 ± 0.00	2.00 ± 0.15
Conj. bilirubin	*µ*mol/L	1.00 ± 0.1	1.00 ± 0.01	1.00 ± 0.10	1.00 ± 0.01
Unconj. bilirubin	*µ*mol/L	2.00 ± 0.01	2.00 ± 0.01	1.00 ± 0.00	1.00 ± 0.00

Values are presented as mean ± SEM (*n* = 10).

^*∗*^Significantly different compared to control at *p* < 0.05.

**Table 6:** Effect of aqueous root extracts of *Dicoma anomala* for 90 days on biochemical parameters of female Wistar rats.

Parameters	Unit	Dose (mg/kg/day)			
		Control	125	250	500
Total cholesterol	mmol/L	2.30 ± 0.23	2.00 ± 0.15	2.20 ± 0.13	2.10 ± 0.20
Triglycerides	mmol/L	0.56 ± 0.00	0.67 ± 0.11	0.85 ± 0.20	0.89 ± 0.01
HDL-c	mmol/L	1.65 ± 0.23	1.15 ± 0.17	1.30 ± 0.25	1.30 ± 0.19
LDL-c	mmol/L	0.80 ± 0.00	0.70 ± 0.11	0.70 ± 0.14	0.60 ± 0.50
ALT	U/L	49.00 ± 0.85	46.00 ± 0.55^*∗*^	45.00 ± 0.77^*∗*^	45.00 ± 0.74^*∗*^
AST	U/L	210.00 ± 0.85	178.00 ± 0.90^*∗*^	150.00 ± 0.87^*∗*^	182.00 ± 0.88^*∗*^
ALP	U/L	165.00 ± 0.87	170.00 ± 0.90^*∗*^	235.00 ± 0.47^*∗*^	210.00 ± 0.75^*∗*^
Glucose	mg/dL	3.23 ± 0.10	3.10 ± 0.11	4.29 ± 0.05	4.74 ± 0.23
Sodium	mmol/L	107.00 ± 0.67	115.00 ± 0.85^*∗*^	123.00 ± 0.44^*∗*^	144.00 ± 0.53^*∗*^
Potassium	mmol/L	5.49 ± 0.01	5.29 ± 0.01	4.99 ± 0.02	5.61 ± 0.00
Chloride	mmol/L	107.00 ± 0.11	106.00 ± 0.21	107.00 ± 0.12	107.00 ± 0.01
Calcium	mmol/L	2.32 ± 0.67	2.30 ± 0.10	2.31 ± 0.14	2.33 ± 0.20
Magnesium	mmol/L	0.80 ± 0.40	0.82 ± 0.02	0.85 ± 0.14	0.88 ± 0.01
Urea	mmol/L	7.99 ± 0.01	7.65 ± 0.00	7.89 ± 0.12	8.05 ± 0.24
Creatinine	*µ*mol/L	43.00 ± 0.23	40.00 ± 0.21^*∗*^	40.00 ± 0.17^*∗*^	37.00 ± 0.20^*∗*^
Albumin	g/L	35.00 ± 0.15	33.00 ± 0.20	32.00 ± 0.10^*∗*^	34.00 ± 0.05
Total protein	g/L	65.00 ± 0.3	65.00 ± 0.25	65.00 ± 0.33	64.00 ± 2.0
Total bilirubin	*µ*mol/L	2.00 ± 0.11	2.00 ± 0.12	3.00 ± 0.00	3.00 ± 0.15
Conj. bilirubin	*µ*mol/L	1.00 ± 0.1	1.00 ± 0.01	1.00 ± 0.10	1.00 ± 0.01
Unconj. bilirubin	*µ*mol/L	2.00 ± 0.01	2.00 ± 0.01	1.00 ± 0.00	1.00 ± 0.00

Values are presented as mean ± SEM (*n* = 10).

^*∗*^Significantly different compared to control at *p* < 0.05.

**Table 7:** Reported biological activities of the identified bioactive compounds from *D. anomala*.

Compound	% Composition	Biological activity
5-(Hydroxymethyl)-2-furancarboxyaldehyde [C_6_H_6_O_3_]	40.64	Antimicrobial preservative, antifungal [10], anti-inflammatory [11–13], antihepatotoxic [14] activity
2,3-Dihydro-3,5-dihydroxyl-6-methyl-4H-pyran-4-one [C_11_H_8_O_4_]	1.52	Antimicrobial, anti-inflammatory activity [15]
n-Tridecane [C_13_H_28_]	5.56	Antimicrobial activity [16]
1,6-Anhydro-*β*-D-glucopyranoside [C_6_H_10_O_5_]	3.48	Nontoxic on cell-cell communication systems [17]
2-(1-methyl-2,5-dioxoimidazolidin-4-yl) acetic acid [C_6_H_8_N_2_O_4_]	10.90	Nontoxic, antihepatotoxic activity [18]
Acetamide [C_4_H_9_NO_2_]	1.85	Anticonvulsant activity [19]
Hexadecanoic acid [C_16_H_32_O_2_]	4.36	Antioxidant, hypocholesteroemic, nematicide, pesticide, lubricant, antiandrogenic, flavour, haemolytic 5-*α*-reductase inhibitor [15]
9-Octadecenoic acid [C_57_H_104_O_6_]	4.46	Antioxidant, anticancer [20, 21]
Globulol [C_15_H_26_O]	1.68	Antimicrobial [22, 23], antibacterial, antiglutamatergic, sedative activity [24]
Phosphine [C_18_H_15_P]	4.69	Antihypertensive, diuretic, uric acid excretion stimulant, saluretic activity [25]
